# MZPAQ: a FASTQ data compression tool

**DOI:** 10.1186/s13029-019-0073-5

**Published:** 2019-06-03

**Authors:** Achraf El Allali, Mariam Arshad

**Affiliations:** Department of Computer Science, College of computer and Information Sciences, King Saud University, Riyadh, Saudi Arabia

**Keywords:** DNA compression, Next generation sequences, FASTA files, FASTQ files

## Abstract

**Background:**

Due to the technological progress in Next Generation Sequencing (NGS), the amount of genomic data that is produced daily has seen a tremendous increase. This increase has shifted the bottleneck of genomic projects from sequencing to computation and specifically storing, managing and analyzing the large amount of NGS data. Compression tools can reduce the physical storage used to save large amount of genomic data as well as the bandwidth used to transfer this data. Recently, DNA sequence compression has gained much attention among researchers.

**Results:**

In this paper, we study different techniques and algorithms used to compress genomic data. Most of these techniques take advantage of some properties that are unique to DNA sequences in order to improve the compression rate, and usually perform better than general-purpose compressors. By exploring the performance of available algorithms, we produce a powerful compression tool for NGS data called MZPAQ. Results show that MZPAQ outperforms state-of-the-art tools on all benchmark datasets obtained from a recent survey in terms of compression ratio. MZPAQ offers the best compression ratios regardless of the sequencing platform or the size of the data.

**Conclusions:**

Currently, MZPAQ’s strength is its higher compression ratio as well as its compatibility with all major sequencing platforms. MZPAQ is more suitable when the size of compressed data is crucial, such as long-term storage and data transfer. More efforts will be made in the future to target other aspects such as compression speed and memory utilization.

## Background

The unit of sequencing data has shifted from “Megabase” to “Terabase” due to a remarkable drop in sequencing cost. Researchers generally have to maintain huge amount of raw genomic data. Therefore, they require efficient ways of storing, transferring and accessing this data. The flood of NGS data from various genomic and metagenomic projects is expected to increase as further progress is made in high throughput sequencing technologies (HTS). Because of the high cost of storing raw sequence data, it is usually pre-processed; analyzed and only conclusions of the studies are saved. In addition, large amount of raw data remain local and never shared due to the high bandwith cost, which affects the knowledge that can be gained from sequencing projects. This has become a major bottleneck in computational biology, as the cost of maintaining the genomic data is exceeding the cost of sequencing it. Currently, biologists are using multi-purpose compression tools that are not designed for biological data and do not take advantage of the nature of the data to achieve greater compression. Though specific compression algorithms are being designed for genomic data, they are either unavailable as a tool or do not perform uniformly on all platforms or different data sizes.

Typically, NGS data is stored either in FASTA or FASTQ format. FASTA is a commonly used text-based format that represents nucleotide sequences. The format includes a line for sequence identification followed by the sequence in a separate line. FASTA allows for multiple biological sequences to be stored in the same file. FASTQ files allow for the inclusion of more information by adding two more lines: one for optional identification information and the other for quality scores for each base in the sequence. Similarly, FASTQ allows multiple sequences to be stored in the same file, which makes it ideal for raw NGS sequencing data.

Several improvements have been made since the first ever DNA compression algorithm was introduced in 1993. Evidence suggests that while the major milestones in compression have been reached, more progress is still needed. Recent survey suggests that there is no single algorithm that works best on all types and sizes of data [1]. In this paper, we investigate the performance of selected state-of-the-art compression algorithms on biological sequences, identification information and quality scores. The idea is to select the best performing algorithm for each sub-stream (line) of FASTQ files, whether it is a generic algorithm, purpose specific or even part of a more complex compression algorithm. By combining the best performing algorithms for most or all of the benchmark datasets, we produce a compression tool that provides the best compression ratios for FASTQ files when compared to state-of-the-art compression tools. We have selected the most prominent state-of-the-art approaches for FASTA and FASTQ compression along with the main general-purpose compression algorithms. Only tools that support non-ACTG characters were considered to ensure they can be used for raw data. Algorithms that do not have publicly available source code or binaries were excluded as well.

Two of the selected tools compress FASTA files only. The first one is called Deliminate [2]. It implements an efficient lossless compression algorithm that combines Delta encoding and progressive elimination of nucleotide characters method. Delta encoding is used to encode the position of the two most frequent nucleotide bases and binary encoding is used for the other two bases. Finally, 7-Zip is used to create an archive of all generated files. The second algorithms is called MFCompress [3] and is one of the most efficient lossless non-referential compression algorithms available for compression of FASTA files according to recent survey [4]. It employs finite-context models for compression of both fields in FASTA files. The identification lines are compressed using single-finite context models, while sequences are encoded using competing multiple finite-context models as well as arithmetic coding.

For FASTQ files, we selected the top four algorithms that meet our criteria. The first one is called SCALCE [5]. It is mainly a boosting scheme that uses Locally Consistent Parsing technique for compression of FASTQ sequences. It rearranges the reads in a way that offers high compression rate and speed, without using a reference genome and irrespective of the compression algorithm used [5]. SCALCE compresses quality scores using Order-3 Arithmetic coding, while compression of identification information is done by gzip, taking into consideration the reordering of reads provided by SCALCE. Results show significant improvement in the compression rate and running time as compared to running the underlining algorithms on unordered reads.

Leon [6] is another FASTQ compression tools. It constructs a de Bruijn graph *G* from the FASTQ reads and encodes each read as a part within *G*. To avoid the memory overhead of the de Bruijn graph, Leon exploits Bloom filter [7] to store the nodes of the graph. Leon encodes a starting k-mer for each read as well as read’s branching information in the graph. The encoded information is compressed using order-0 arithmetic coding. For quality scores, Leon employs zlib and supports both lossy and lossless compression.

The last two algorithms we used in this study are LFQC [8] and Slimfastq [9]. LFQC is a lossless compression scheme developed for compression of FASTQ files. The key contribution is its advanced read-identifier tokenization scheme. It uses PAQ family members for compression of read sequences and quality scores. IPAQ is used for compression of reads while ZPAQ is used for compression of quality scores. Slimfastq is a robust re-implementation of another FASTQ algorithm Fqzcomp [10]. It is one of the fastest FASTQ compression algorithms that provides reasonable compression rates.

We also considered three of the most commonly used general-purpose compression tools that work for genomic data. We used these tools to compress different streams in FASTQ files and compared them in combination with FASTA compression algorithms. These algorithms serve as baseline comparison of the domain specific compression tools. The first algorithm is gzip, which is a general-purpose compression algorithm that combines Huffman coding and LZ77 to construct a dictionary that is optimized according to repetitions of words in the data. It offers the fastest compression and decompression speeds with minimal memory requirements among all general-purpose compressors used in this study. Bzip2 is another compression scheme that uses Burrows-Wheeler transform along with Huffman coding compression. The symbols within the input data are relocated to increase repetitions of a particular sub-sequence, which can be encoded more efficiently based on their probability of occurrence. Generally, bzip2 offers better compression than gzip [11]. The third algorithm used in this study is LZMA. It employs an optimized version of the Lempel-Ziv-Markov algorithm (LZ77) [12]. LZMA makes use of large dictionary sizes and provides special support for repeatedly used match distances. It provides better compression than LZ77 by utilizing a history buffer, smaller codes for recent repeats, a sophisticated dictionary data structure and an optimal arithmetic coding scheme selected by dynamic programming. LZMA has better compression-ratio than gzip and bzip2 but such an improvement comes at the cost of memory and time [8]. Table 1 summarizes the characteristics of the tools used in this study.

**Table 1:** Characteristics of selected compression

	Input	C-ratio	Speed	Memory	Techniques
gzip	General ASCII	Moderate	High	Low	LZ77 and Huffman coding
bzip2	General ASCII	Moderate	High	Low	BWT and Huffman coding
LZMA	General ASCII	Moderate	Low	High	Lempel-Ziv Markov chain and LZ77
Deliminate	FASTA	High	High	Low	Delta encoding with Lempel-Ziv
MFCompress	FASTA	High	Moderate	High	Finite Contexts Models
Leon	FASTQ	High	High	Moderate	De Bruijn graph and Order-0 Arithmetic coding
Slimfast	FASTQ	High	High	Moderate	Delta encoding, Arithmetic coding, and Context Models
SCALCE	FASTQ	High	High	High	Reordering, gzip, bzip2 and Order-3 Arithmetic coding
LFQC	FASTQ	High	Low	High	PAQ compressors

## Methods

### Datasets

We use a set of compression benchmark datasets that were recently compiled by the MPEG (Moving Picture Expert Group) HTS compression working group [1]. The dataset was developed to allow accurate and fair evaluation of compression tools. The benchmark also allows for reproduction of the evaluation process [1]. The actual size of the MPEG benchmark dataset is 2.4 TB, of which a subset (85 GB) is publicly available and is used in this study. The dataset has a wide range of characteristics: it covers leading sequencing platforms (Illumina, Pacific Biosciences); it includes deep and shallow coverage, both fixed-length and variable-length reads and different organisms (Homo sapiens, bacteria, plant); it also includes datasets of varying sizes (0.5 GB - 53 GB). More details of these datasets are shown in Table 2.

**Table 2:** Description of benchmark datasets

Identifier	Size (MB)	Type	Technique	Organism	Description
SRR 554369	456	FASTQ paired short reads	Illumina GAIIx; 50x total depth	P.aeruginosa	Small genome (6-7 MB), medium depth
SRR 327342	3,881	FASTQ paired short reads	Illumina GAII; 175x total depth;	S.cerevisiae	Small genome (12 MB), high depth.
MH0001. 081026	1,880	FASTQ paired short reads	Illumina GA; unknown depth	Human gut metagenome	Mixed species and unknown references
SRR 1284073	1,309	FASTQ single variable-length long reads	PacBio; 140x depth	Bacteria E.Coli	Small genome (4.7 MB), higher error rate.
SRR 870667	22,987	FASTQ paired short reads	Illumina GAIIx; 35x total depth	Plant T.cacao.	Medium sized genome (345 MB)
ERR 174310	53,869	FASTQ paired short reads	Illumina HiSeq 2000; 13x total depth	H.sapiens (NA12877) individual	Common instrument depth

### Methodology

**Fig. 1:**
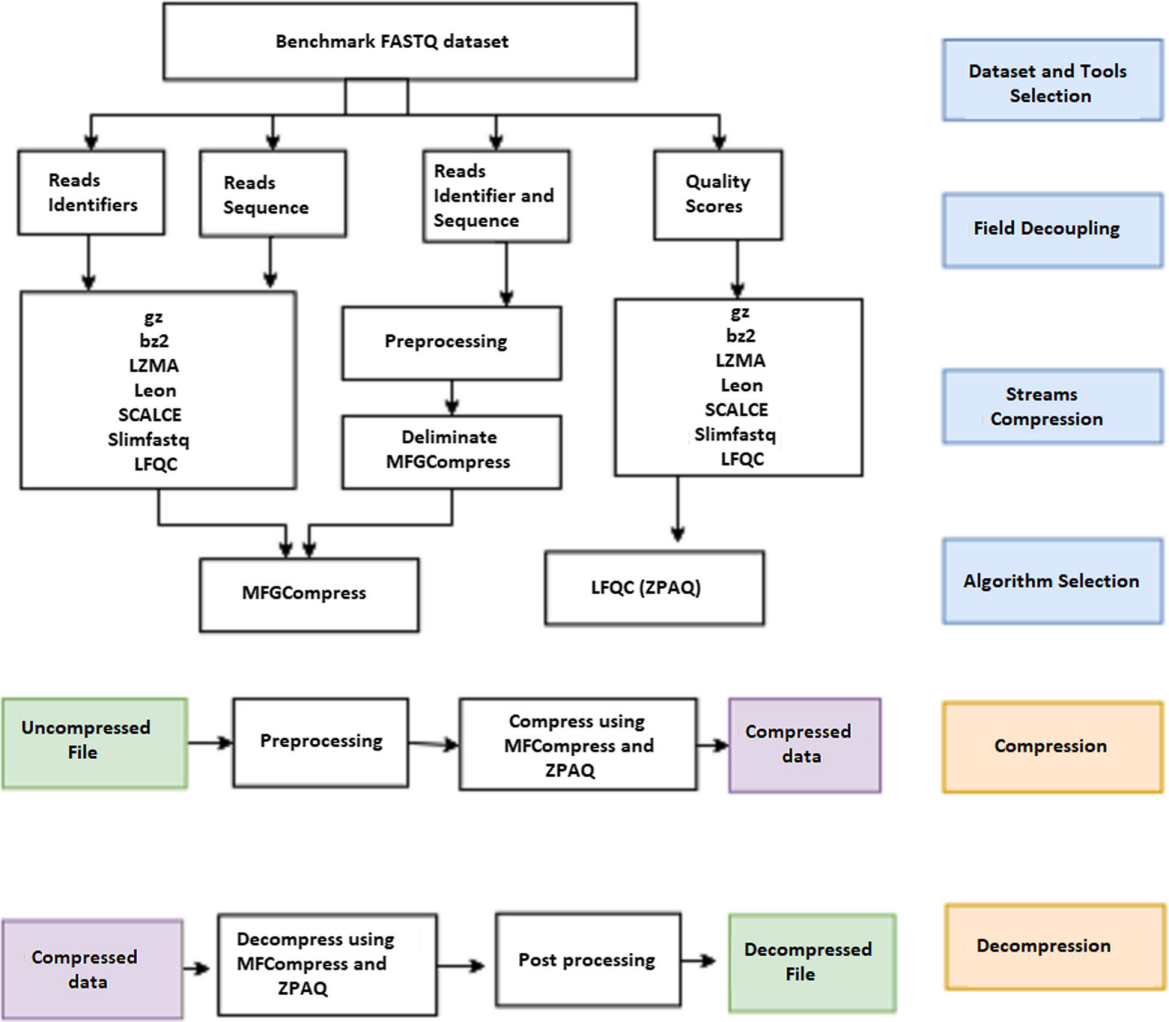
MZPAQ: Illustration of the overall framework used to obtain MZPAQ

Our goal is to produce a FASTQ compression tool that produces the best compression ratio regardless of the type and size of the dataset. Therefore, we investigate the compression ratio of the best algorithms reported by recent surveys. We first split the content of FASTQ data into different streams (field decoupling) and compress each stream using all compression algorithms that support that stream (stream compression). We then compare the performance of each tool for an individual stream and select the algorithms that perform best or second to best on all datasets in the benchmark (algorithm selection). Finally, we combine the selected algorithms and sub-algorithms in order to create a tool that provides better compression ratios for FASTQ files of different characteristics (compression and decompression). The framework used in this study is illustrated in Fig. 1.

Based on the obtained results, we selected the most promissing techniques as candidates (i.e. the algorithms or sub-algorithm that produced the highest compression ratio for most of the datasets in the benchmark). For compression of read identifiers and sequences, we found MFCompress to be the most appropriate since it works for all types of FASTQ input datasets and offers the best or second to best compression ratios. For quality scores, LFQC offers the best compression ratio for all datasets. Thus, we selected the sub-algorithm used by LFQC to compress quality scores, which is ZPAQ [8]. Complete results and evidence supporting the selection of MFCompress and ZPAQ are presented in the “Results” section.

Our tool is a hybrid of MFCompress (v 1.01) and ZPAQ (v 7.15), hence the name MZPAQ. In order to compress a FASTQ file, MZPAQ scans the input file and divides it into the four streams of FASTQ format. The first two streams (i.e. read identifier and read sequence) are compressed using MFCompress after the identifier stream is pre-processed to comply with the format restrictions of MFCompress. The third stream is discarded during compression as it contains a ’+’ symbol followed by an optional comment similar to identifier field which can be regenerated later at the time of decompression [13]. This is similar to all available tools including those used for comparison in this study. The fourth stream is compressed using the strong context-mixing algorithm ZPAQ. The output from each stream are then combined in a single binary file.

The decompression module of MZPAQ performs the inverse of the compression. The identifiers and reads are decompressed using MFCompress and ZPAQ is used to decode compressed quality scores. This results into two uncompressed data sources. After decompression, the third stream of FASTQ data is regenerated and all uncompressed data sources are combined and arranged to generate the FASTQ output file.

## Results

In this section, we present the compression results for different streams using state-of-the-art and general purpose tools. We then show the performance comparison between our approach and the other tools. The performance is presented in terms of compression-ratio, compression speed and memory usage. We also evaluate the ability of each tool to correctly compress the benchmark datasets.

### Compression of FASTQ streams

#### Compression of identifiers and sequences

Read identifiers are typically platform specific. In many cases, read identifiers contain instrumental information in addition to their unique information, which makes identifiers more compressible than sequences and quality scores. FASTQ sequences are strings of the alphabet A, C, T and G and occasionally N for unknown bases. In order to select the best technique for these two streams, we used general purpose and FASTQ compression tools to compress the identifiers and sequence streams. Moreover, we used FASTA tools, namely Deliminate and MFCompress, on these streams. Since FASTA compression tools do not output individual compressed streams, we looked at the compression ratios for identifier and sequence fields collectively. Table 3 shows a comparison of identifier and sequence compression using the benchmark datasets.

**Table 3:** Compression of identifiers and sequences: Blue color represents original file size

Dataset	Method	Read identifiers (MB)	Sequences (MB)	Identifiers & Sequences (MB)	C-ratio
SRR554369	Original	blue60.8	blue167.4	blue228.2	
	gzip	7.5	48.8	56.3	4.05
	bzip2	3.8	46.7	50.5	4.52
	LZMA	0.8	17.6	18.4	12.40
	Leon	0.1	18.6	18.7	12.20
	SCALCE	6.8	17.0	23.8	9.59
	Slimfastq	0.1	29.9	30.0	7.61
	LFQC	0.0	17.4	17.4	green13.15
	Deliminate	N/A	N/A	27.9	8.18
	MFCompress	N/A	N/A	14.0	**16.30**
SRR327342	Original	blue978.1	blue962.3	blue1940.4	
	gzip	83.6	284.0	367.6	5.28
	bzip2	70.9	269.4	340.3	5.70
	LZMA	46.7	120.2	166.9	11.63
	Leon	26.3	89.3	115.6	**16.79**
	SCALCE	69.7	68.4	138.1	14.05
	Slimfastq	23.0	149.0	172.0	11.28
	LFQC	20.9	128.7	149.6	12.97
	Deliminate	N/A	N/A	164.1	11.83
	MFCompress	N/A	N/A	124.9	green15.54
MH0001	Original	blue416.4	blue523.8	blue940.2	
	gzip	36.7	157.5	194.2	4.84
	bzip2	32.1	151.4	183.5	5.12
	LZMA	22.0	101.7	123.7	7.60
	Leon	20.6	87.0	107.6	**8.74**
	SCALCE	76.7	70.7	147.4	6.38
	Slimfastq	17.5	103.7	121.2	7.76
	LFQC	16.1	103.2	119.3	7.88
	Deliminate	N/A	N/A	115.9	8.11
	MFCompress	N/A	N/A	111.1	green8.46
SRR1284073	Original	blue4.9	blue649.6	blue654.5	
	gzip	0.8	182.7	183.5	3.57
	bzip2	0.7	176.5	177.2	3.69
	LZMA	0.5	160.1	160.6	4.08
	Leon	0.3	170.1	170.4	3.84
	SCALCE	N/A	N/A	N/A	N/A
	Slimfastq	N/A	N/A	N/A	N/A
	LFQC	0.3	155.8	156.1	4.19
	Deliminate	N/A	N/A	155.2	**4.22**
	MFCompress	N/A	N/A	155.9	green4.20
SRR870667	Original	blue3,947.2	blue7.546.1	blue11,493.3	
	gzip	514.3	2081.6	2595.9	4.43
	bzip2	422.0	1,974.4	2396.4	4.80
	LZMA	280.4	1515.7	1796.1	6.40
	Leon	139.7	1363.1	1502.8	7.65
	SCALCE	341.6	999.4	1341.0	**8.57**
	Slimfastq	128.2	1419.1	1547.3	7.43
	LFQC	122.2	N/A	N/A	N/A
	Deliminate	N/A	N/A	1768.5	6.50
	MFCompress	N/A	N/A	1407.7	green8.16
ERR174310	Original	blue11,107.5	blue21,173.1	blue32,280.6	
	gzip	1483.8	6018.7	7501.7	4.30
	bzip2	1223.6	5745.2	6968.8	4.63
	LZMA	691.0	4982.0	5673.0	5.69
	Leon	355.2	4734.4	5089.6	6.34
	SCALCE	1073.0	3016.0	4089.0	**7.89**
	Slimfastq	323.4	4426.4	4749.8	6.80
	LFQC	N/A	N/A	N/A.0	N/A
	Deliminate	N/A	N/A	5604.0	5.76
	MFCompress	N/A	N/A	4666.3	green6.92

Best results are bold faced and second to best are colored green. N/A refers to unsupported or unsuccessful cases

From the results, we observe that compression ratios for identifier and sequence streams are highly variable (from 4:1 to 16:1). Gzip, bzip2, LZMA and Slimfastq did not give best or second to best result for all datasets. Leon and SCALCE each performed best on two of the datasets. Deliminate gave best compression ratios for one dataset and LFQC gave the second to best ratio for one dataset. Most importantly, we notice that MFCompress has the best ratio for the first dataset and second to best for all other benchmark datasets.

Gzip, bzip2, LZMA, Leon, Deliminate and MFCompress are able to compress all the datasets while SCALCE and Slimfastq did not work for the PacBio dataset and LFQC did not give results in two cases. Since the main goal of our study is to develop a compression scheme that works and performs best for all data types, and based on the above findings, we select MFCompress as it works for all datasets while producing best or second to best compression ratios.

#### Compression of quality scores

Quality scores are ASCII characters with larger alphabet size than read sequences, which makes them more difficult to compress. Each quality score has a strong correlation with a number of preceding quality scores. This correlation decreases as the distance between two quality scores increases. Furthermore, the rate of change of correlation randomly changes from one FASTQ file to another [9]. These characteristics make it challenging to code quality scores efficiently for all datasets. Therefore, the compression ratios for quality score streams are less than those of the read identifiers and sequences. Table 4 shows the performance comparison of different algorithms on quality scores. The compression ratios for quality scores is between 2:1 and 4:1. Slimfastq gives the second to best ratio for all datasets except for the PacBio dataset, for which it does not work. The results clearly indicate that LFQC is the best suitable candidate for compressing quality scores as it gives the best compression ratios for all datasets.

**Table 4:** Compression of Quality Scores: Blue color represents original file size

Dataset	Method	Compression size (MB)	Compression ratio
SRR554369	Original	blue167.4	
	gzip	64.7	2.59
	bzip2	57.6	2.91
	LZMA	57.0	2.94
	Leon	64.6	2.59
	SCALCE	52.0	3.22
	Slimfastq	green47.8	green3.50
	LFQC	**47.6**	**3.52**
SRR327342	Original	blue962.3	
	gzip	428.6	2.25
	bzip2	405.8	2.37
	LZMA	383.5	2.51
	Leon	429.1	2.24
	SCALCE	349.3	2.75
	Slimfastq	green334.9	green2.87
	LFQC	**332.0**	**2.89**
MH0001	Original	blue523.8	
	gzip	184.4	2.84
	bzip2	173.5	3.02
	LZMA	165.9	3.16
	Leon	183.9	2.85
	SCALCE	297.5	1.76
	Slimfastq	green144.8	green3.62
	LFQC	**142.3**	**3.68**
SRR1284073	Original	blue649.6	
	gzip	308.7	2.10
	bzip2	283.6	2.29
	LZMA	green280.5	green2.32
	Leon	308.6	2.10
	SCALCE	N/A	N/A
	Slimfastq	N/A	N/A
	LFQC	**250.7**	**2.59**
SRR870667	Original	blue7,546.1	
	gzip	3021.5	2.50
	bzip2	2780.7	2.71
	LZMA	2668.8	2.83
	Leon	3022.4	2.50
	SCALCE	2365.0	3.19
	Slimfastq	green2281.7	green3.31
	LFQC	**2259.9**	**3.34**
ERR174310	Original	blue21,173.1	
	gzip	8525.8	2.48
	bzip2	7439.9	2.85
	LZMA	7397.0	2.86
	Leon	8533.3	2.48
	SCALCE	6738.0	3.14
	Slimfastq	green6295.0	green3.36
	LFQC	**6103.0**	**3.47**

Best results are bold faced and second to best are colored green. N/A refers to unsuccessful cases

### MZPAQ compression performance

In this section, we compare the performance of MZPAQ against several state-of-the-art FASTQ compression tools as well as general-purpose compression tools. The methods are compared based on compression ratio, compression speed and memory usage during compression. The comparison also includes the ability of the tool to produce exact replica of the original file after decompression.

#### Compression ratio

The ratio between the size of the original and the compressed files is calculated for each dataset using all the compression tools. Table 5 shows the performance of MZPAQ relative to other evaluated tools in terms of compression ratio. The results clearly indicate that MZPAQ achieves the highest compression ratios compared to all the other tools for all datasets. LFQC achieves the second to best compression ratios for smaller file sizes; however, it does not work for larger datasets. All domain-specific tools performed better than general-purpose tools, except for LZMA, which did not work on PacBio data.

**Table 5:** Compression ratios of evaluated tools

Dataset	SRR554369	SRR327342	MH0001	SRR1284073	SRR870667	ERR174310
Gzip	3.16	3.87	3.89	2.40	3.43	2.96
Bzip2	3.74	4.67	4.82	2.83	4.06	3.62
LZMA	4.99	5.47	5.15	2.84	4.40	3.67
Leon	5.48	7.13	6.45	2.73	5.08	3.95
SCALCE	5.97	7.96	6.32	N/A	6.20	4.98
Slimfastq	5.87	7.66	7.07	N/A	6.00	4.88
LFQC	7.02	8.06	7.18	**3.22**	N/A	N/A
MZPAQ	**7.04**	**8.49**	**7.98**	**3.22**	**6.27**	**5.00**

N/A refers to unsuccessful compression

The values in bold typeface represent the best performance

#### Compression speed

Compression speed is the number of compressed MB per second. The decompression speed is computed similarly. In order to conduct the comparison, we run all the tools in single thread mode to allow for direct comparison between all the tools, as some of them do not support multi-threading. Table 6 shows the compression speed performance of the compared algorithms in MB/s. Slimfastq is the fastest tool and provides maximum compression speed for all cases except in the case of PacBio data, which it does not support. LFQC is the slowest for all the datasets it supports. In case of decompression speed. We can see from the results shown in Table 7 that gzip outperformes all the evaluated tools, decompressing at over 45 MB per second for all datasets. We further notice that general-purpose tools have faster decompression than compression speeds, particularly LZMA. While faster compression/decompression is favorable, the speed may be achieved at the cost of the compression ratio.

**Table 6:** Compression Speed of evaluated tools

Dataset	SRR554369	SRR327342	MH0001	SRR1284073	SRR870667	ERR174310
Gzip	5.77	11.22	4.69	5.13	6.18	6.24
Bzip2	14.71	12.48	12.96	11.48	12.91	12.41
LZMA	0.91	1.24	1.05	0.79	1.04	0.96
Leon	3.86	5.94	4.8	3.54	3.12	3.35
SCALCE	18.24	21.8	19.58	N/A	12.95	9.24
Slimfastq	**38**	**49.76**	**45.85**	N/A	**40.76**	**33.92**
LFQC	0.82	0.98	1.21	0.7	N/A	N/A
MZPAQ	0.98	1.34	1.33	0.78	0.99	0.83

N/A refers to unsuccessful compression

The values in bold typeface represent the best performance

**Table 7:** Decompression speed of evaluated tools

Dataset	SRR554369	SRR327342	MH0001	SRR1284073	SRR870667	ERR174310
Gzip	**152**	**110.89**	**144.62**	**145.44**	**48.09**	**46.84**
Bzip2	35.08	32.07	33.57	22.96	24.48	22.03
LZMA	76	55.44	62.67	46.75	35.47	33.77
Leon	16.29	18.39	27.65	8.5	13.12	9.26
SCALCE	25.33	31.55	27.24	N/A	22.3	19.12
Slimfastq	24	24.72	20.89	N/A	20.58	17.25
LFQC	0.8	1.04	1.11	0.68	N/A	N/A
MZPAQ	0.91	1.07	1.29	0.82	0.97	0.99

N/A refers to unsuccessful compression

The values in bold typeface represent the best performance

#### Memory usage

Memory usage refers to the maximum number of memory bytes required by an algorithm during compression or decompression, it represents the minimum memory that should be available for successful execution of a program. In general, memory usage varies with the type of datasets. Tables 8 and 9 show the maximum memory requirements for compression and decompression, respectively. The results show that LZMA requires 10 times more memory for compression as compared to decompression. Leon uses almost two times more memory for compression than decompression. In all cases, gzip requires the least amount of memory.

**Table 8:** Compression memory usage of evaluated tools

Dataset	SRR554369	SRR327342	MH0001	SRR1284073	SRR870667	ERR174310
Gzip	**1.8**	**1.9**	**1.8**	**1.9**	**1.8**	**1.9**
Bzip2	7.8	8.7	8.8	8.3	7.7	8.7
LZMA	691.4	691.5	691.3	691.4	691.4	691.3
Leon	382.2	385.7	95.1	4213.5	1858	3324.6
SCALCE	1429.9	3111.2	2584.2	N/A	5424.5	5450.4
Slimfastq	82.5	82.5	82.5	N/A	82.5	82.6
LFQC	1445.2	1189.5	1540.9	1522	N/A	N/A
MZPAQ	2398.8	2901.8	2691	2385.6	4544.5	5326.4

N/A refers to unsuccessful compression

The values in bold typeface represent the best performance

**Table 9:** Decompression memory usage of evaluated tools

Dataset	SRR554369	SRR327342	MH0001	SRR1284073	SRR870667	ERR174310
Gzip	**1.7**	**1.6**	**1.6**	**1.6**	**1.6**	**1.8**
Bzip2	5	5	5	4.8	4.9	4.9
LZMA	67.8	67.8	67.7	67.7	67.8	67.8
Leon	247.8	221.07	35.3	2923.9	762.2	2971
SCALCE	1031.4	1030.6	1031.1	N/A	1031.1	1032.6
Slimfastq	82.5	82.5	82.4	N/A	82.2	82.3
LFQC	1457.7	1559.6	1451.6	1527.7	N/A	N/A
MZPAQ	2383.9	2382	2384.1	2383	2396.3	2383

N/A refers to unsuccessful compression

The values in bold typeface represent the best performance

## Discussion

Evaluating the effectiveness of high-throughput sequencing data compression tools has gained a lot of interest in the last few years [1, 13–15]. Comparative reviews of prominent general-purpose as well as DNA-specific compression algorithms show that DNA compression algorithms tend to compress DNA sequences much better than general-purpose compression algorithms [1, 4]. While FASTA compression tools show promising results, the majority of raw data is saved in FASTQ format for which compression tools are yet to mature and support all types and sizes. For example, Table 10 shows the results of compression for all the benchmark datasets. We can see that all the evaluated compression tools are not able to compress variable-length reads obtained by Pac Bio except for MZPAQ. While LFQC produces results that are comparable and only slightly less than MZPAQ, it does not work for identifier and sequence compression of large datasets.

**Table 10:** Compression of benchmark datasets using FASTQ tools

Dataset	Size (MB)	Leon	SCALCE	Slimfastq	LFQC	MZPAQ
SRR554369	456	$\checkmark $	$\checkmark $	$\checkmark $	$\checkmark $	$\checkmark $
SRR327342	3,881	$\checkmark $	$\checkmark $	$\checkmark $	$\checkmark $	$\checkmark $
MH0001	1,880	$\checkmark $	$\checkmark $	$\checkmark $	$\checkmark $	$\checkmark $
SRR1284073	1,309	yellow ×	orange ×	red ×	orange ×	$\checkmark $
SRR870067	22,987	$\checkmark $	$\checkmark $	$\checkmark $	red ×	$\checkmark $
ER174310	53,869	$\checkmark $	$\checkmark $	$\checkmark $	red ×	$\checkmark $

red ×: Tool does not support data.orange ×: Tool produces invalid output.yellow ×: Tool produces wrong output

In our study, we evaluate various existing efficient algorithms to investigate their ability to compress FASTQ streams. In addition, we evaluate FASTA tools on the identifier and sequence streams of FASTQ files. The reason behind this is the fact that FASTA compression tools have been developed for longer than FASTQ compression tools. Moreover, they have been shown to outperform general purpose tools in compressing identifiers and reads. We selected two FASTA and four FASTQ compression tools that have been reported to offer the best compression ratios by recent surveys. Both FASTA tools successfully compressed identifiers and sequences of all benchmark datasets while some FASTQ tools are not successful on large datasets.

Among the evaluated tools, we select MFCompress for compression of identifier and sequence streams. We also found ZPAQ to be a suitable candidate for compression of quality scores after evaluating all the tools on this stream. A point worth noticing here is that both MFCompress and ZPAQ make use of context modeling, which makes this compression technique very promising for compression of genomic data [16]. Our evaluation illustrates the significant impact on compression efficiency when we divide FASTQ into multiple data streams and use different compression schemes based on the stream type. As a result, we created MZPAQ, which uses MFCompress and ZPAQ as the underlining algorithms in order to deliver better compression ratios for all three main components of FASTQ data.

**Fig. 2:**
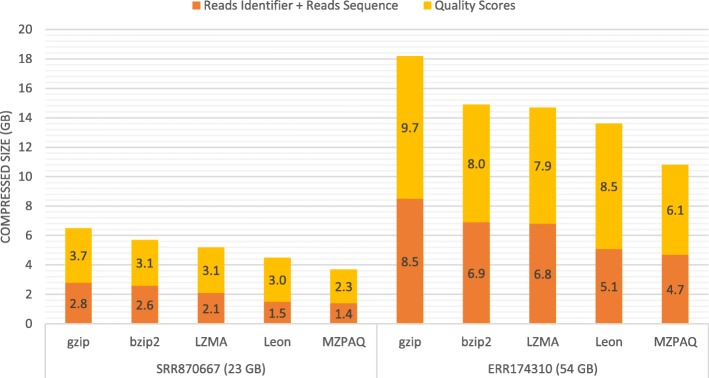
Comparison: Compression sizes of different fastq steams in two large datasets using different compression tools

MZPAQ outperforms existing tools in terms of compression ratios for all types of FASTQ benchmark datasets. In some cases, the compression ratio gain is minor; however, our goal is to create a tool that works best for all types of data. Our evaluation shows that existing tools support only Illumina files containing short and fixed-length reads. These tools are not optimized to support variable-length reads data from the PacBio platform. Other than Leon, MZPAQ is the only domain-specific algorithm that works for all FASTQ datasets. In addition, MZPAQ outperforms the compression ratios of Leon. Figure 2 shows a comparison of different tools that work for all benchmark datasets. The figure shows that MZPAQ outperforms comparable tools for both the combined identifier-sequence stream as well as the quality scores stream. A key observation here is that the compression ratios for quality scores vary from 2:1 to 4:1 while identifier and sequence data compression ratios are in the range of 4:1 to 17:1. It is evident that the nature of quality scores makes it challenging to compress them as compared to other streams of FASTQ data. With general-purpose and domain-specific compression algorithms efficiently compressing identifier and sequences while delivering only moderate compression ratios for quality scores, there is a growing need to develop compression schemes to better compress quality scores [17, 18].

**Fig. 3:**
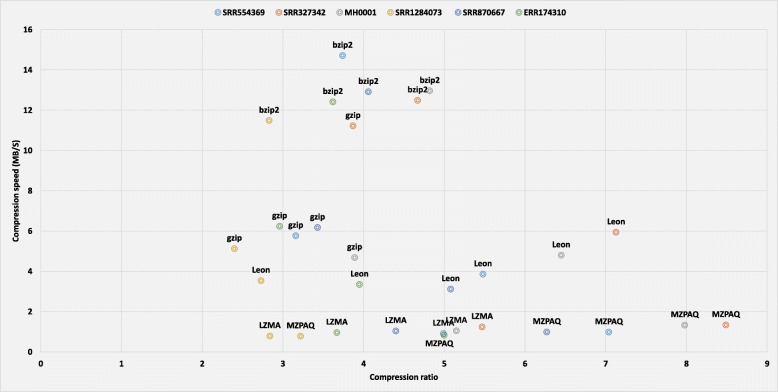
Compression ratio vs. compression speed: The compression ratio versus the speed of compression for all benchmark datasets using different compression tools

**Fig. 4:**
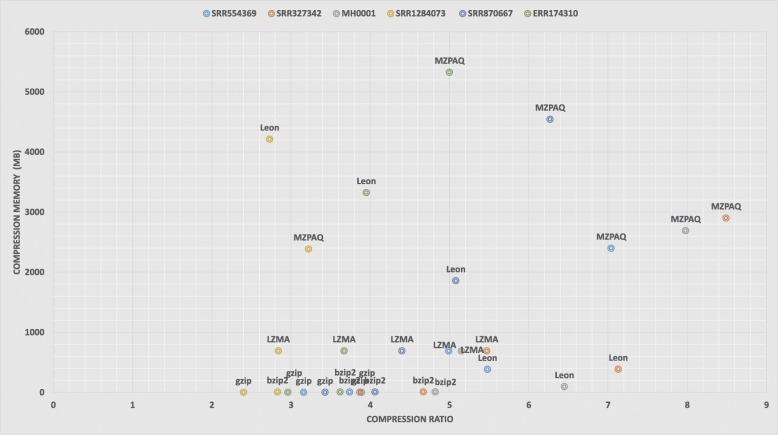
Memory usage vs. compression ratio: The maximum memory used during compression versus the compression ratio for all benchmark datasets using different compression tools

From the experimental results, we can see that the best compression ratio, maximum speed, and minimum memory requirements are competing goals. In general, higher compression ratios are achieved by programs that are slower and have higher memory requirement. In our analysis, general-purpose tools have compression ratios from 2:1 to 5:1, with compression speed of up to 15 MB/s (bzip2) and decompression speed up to 150 MB/s (gzip). In the case of domain-specific tools, compression ratios are in the range of 4:1 to 8:1, reaching up to 46 MB/s compression speed (Slimfastq) and 32 MB/s decompression speed (Scalce). Figures 3 and 4 illustrate the trade-off between compression ratio and the speed and memory usage. For example, gzip offers the lowest compression ratio but has the best performance in case of speed and memory usage. Better compression-ratio tools cost both time and memory but they provide valuable long term space and bandwidth savings. When data size is crucial, these tools are crucial.

Figures 3 and 4 clearly demonstrate that almost all compression algorithms, general or domain-specific, have a trade-off between compression ratio, speed, and memory usage. MZPAQ provides better compression ratios for all platforms, at the cost of higher running time and memory usage. MZPAQ is suitable for areas where the preference is to maximize compression ratio for a long-term storage or faster data transfer. In addition, speed performance can be remarkably enhanced by employing high performance computing. There is evidence supporting a considerable increase in speed of MFCompress and ZPAQ, by exploiting parallelism [13, 19, 20].

Based on our analysis of existing compression algorithms, it is obvious that none of these techniques qualify for the one-size-fits-all approach. There is no compression scheme that provides best results in terms of all evaluation metrics we analyzed. For example, datasets that are not well compressed by one algorithm are efficiently compressed by another. One of the main drawbacks of most algorithms is their compatibility with only specific type of input, greatly restricting their usage by biologists who need to compress different types of data. For example, some tools accept only ACTG, support only fixed read length, or support a subset of platforms.

## Conclusions

The backbone of modern genetics is DNA sequencing. Thanks to recent advances in sequencing technologies, there has been an exponential increase in the speed and amount of DNA sequenced on a daily basis. Thus, the need of storage space is also increasing by an equal rate. This implies that if the same trend persists, the cost of DNA sequencing pipeline will be highly influenced by the storage cost, rather than the sequencing itself. In an attempt to solve this problem, developing efficient compression algorithms is crucial.

In this paper, we present a compression tool for the most commonly used format for raw data, which is FASTQ. We first review recent progress related to DNA compression and explore various compression algorithms. To achieve better compression performance, the input is fragmented to expose different kind of information namely identifier strings, quality scores, sequences and other optional fields. The final objective is achieved by recognizing the statistical properties of every specific kind of information to use an appropriate compression method. We combine existing algorithms and sub-algorithms and achieve the best compression ratios on FASTQ files for all datasets from a recent and well known review. Comparative analysis of existing tools as well as our tool show that MZPAQ is able to better compress data from all types of platforms as well as compress data of different sizes. We can conclude that MZPAQ is more suitable when the size of compressed data is crucial such as long-term storage and data transfer to the cloud.

At this point, we present a method that focuses on improving compression ratio for all types of FASTQ datasets. Later, effort will be made to target other aspects such as compression speed and memory requirements. Parallel implementation and code optimization can be used to overcome the high compression cost of MZPAQ.

## Data Availability

The datasets used in this study are available from the Moving Picture Experts Group (MPEG), from the URL https://github.com/sfu-compbio/compression-benchmark/blob/master/samples.md.
